# Analyzing large Alzheimer's disease cognitive datasets: Considerations and challenges

**DOI:** 10.1002/dad2.12135

**Published:** 2020-12-07

**Authors:** Maura Bellio, Neil P. Oxtoby, Zuzana Walker, Susie Henley, Annemie Ribbens, Ann Blandford, Daniel C. Alexander, Keir X. X. Yong

**Affiliations:** ^1^ UCL Centre for Medical Image Computing (CMIC) Department of Computer Science University College London London UK; ^2^ UCL Interaction Centre (UCLIC) Department of Computer Science University College London London UK; ^3^ Division of Psychiatry University College London London UK; ^4^ Dementia Research Centre Department of Neurodegeneration, National Hospital for Neurology and Neurosurgery UCL Queen Square Institute of Neurology University College London London UK; ^5^ Icometrix Leuven Belgium

**Keywords:** Alzheimer's disease, cognition, cognitive tests, composite scores, data‐sharing initiatives, data‐driven computational models, mild cognitive impairment, predictive models

## Abstract

Recent data‐sharing initiatives of clinical and preclinical Alzheimer's disease (AD) have led to a growing number of non‐clinical researchers analyzing these datasets using modern data‐driven computational methods. Cognitive tests are key components of such datasets, representing the principal clinical tool to establish phenotypes and monitor symptomatic progression. Despite the potential of computational analyses in complementing the clinical understanding of AD, the characteristics and multifactorial nature of cognitive tests are often unfamiliar to computational researchers and other non‐specialist audiences. This perspective paper outlines core features, idiosyncrasies, and applications of cognitive test data. We report tests commonly featured in data‐sharing initiatives, highlight key considerations in their selection and analysis, and provide suggestions to avoid risks of misinterpretation. Ultimately, the greater transparency of cognitive measures will maximize insights offered in AD, particularly regarding understanding the extent and basis of AD phenotypic heterogeneity.

## 1 INTRODUCTION

Longitudinal data collection and sharing initiatives represent a step change for research into Alzheimer's disease (AD) progression. Such initiatives have enabled availability of multiple big datasets, which has increasingly encouraged non‐clinical researchers who are developing innovative data‐driven methods to study early detection and progression patterns of the disease.^1^ Cognitive assessments comprise a key component of these datasets. They are widely used in clinical practice, and are considered a primary index for characterizing disease severity and clinical presentation, and a gateway for further investigations.^2^ Moreover, they define clinical understanding of patients’ needs and management, based on cognitive phenotype.

The application and development of cognitive tests is a key aspect of clinical research into improving diagnosis, characterization of samples and longitudinal change, outcome measures including composite scores,^3^ and for validating potential AD biomarkers and data‐driven subtypes. Investigating how cognitive phenotype is associated with genetic, demographic, and anatomical characteristics carries various mechanistic implications for our understanding of AD. Salient questions include to what extent apolipoprotein E (APOE) genotype and other factors influence the considerable phenotypic heterogeneity evident in AD,^2^ ranging from typical memory‐led AD to canonical atypical clinical phenotypes including visual‐/spatial,^4^ language‐, motor‐, or executive‐led presentations, and understanding factors associated with cognitive resilience.

Big data collection initiatives offer an unparalleled opportunity to advance these research areas. There are, however, frequent inconsistencies and misconceptions in the use of neurocognitive data. Common methodological and analytical mistakes include overinterpreting the correspondence between an individual test and a specific cognitive domain or function,^5^ inappropriate definition of “impairment” based on normative data,^6^ and underappreciation of test properties, such as susceptibility to practice, ceiling, and floor effects.^7^ Compounding these are the diversity of cognitive domains, AD presentation (typical, atypical) and progression (preclinical, prodromal, syndromic), the enormous array of tests, and their properties and idiosyncrasies.

This position paper aims to present common pitfalls and promote best practices for data‐driven computational analyses of cognitive measures to maximize their value in the global efforts to understand and manage AD. We highlight key challenges and common pitfalls through examples using cognitive tests commonly available in open access AD datasets.

## 2 BACKGROUND

### 2.1 Cognitive testing

Cognitive tests are used near‐ubiquitously to understand the impact of neurodegenerative disease on patients.^2^ Standardized cognitive tests aim to measure impairment objectively, adjusting for demographic factors that could independently impact scores, minimizing use of subjective and self‐reported measures, while being relatively cheap, widely available for English‐speaking countries, quick to administer, minimally invasive, and with quantifiable reliability for their use in clinical work. A complete assessment is typically composed of several tasks, each intended to examine a broad function or domain, such as memory, attention, executive function, language, and visuospatial processing. Cognitive domains can also be conceptualized in the context of altered function and/or structure of particular brain regions or networks. Additional information can come from behavioral observations and qualitative evaluation. It is not possible to completely isolate measurements for individual domains, as correspondence is limited between individual tests and cognitive function and impairment is multifactorial (eg, poor memory might be attributable to impaired attention or visual processing, rather than a primary memory deficit). In clinical practice it is therefore vital that individual tests scores are always interpreted within the context of an individual patient's overall profile,^2^ rather than in isolation.

**HIGHLIGHTS**

-  Wider availability of Alzheimer's disease shared datasets has stimulated the development of data‐driven approaches to characterize disease progression.
-  Cognitive tests are a key component of such datasets, though their heterogeneous and multifactorial characteristics challenge their deployment in data‐driven computational models.
-  We summarize fundamental properties of cognitive assessments and considerations for informed handling of cognitive data to promote valid analysis and interpretation by non‐specialist researchers.

**RESEARCH IN CONTEXT**

1. **Systematic review**: Increased availability of large biomarker datasets from studies of Alzheimer's disease (AD) has stimulated analytic approaches to understand its characteristics and progression. Cognitive tests both feature in such datasets and are near‐ubiquitous in clinical practice to assess the nature and extent of impairment. The authors reviewed the literature using traditional sources, citing recent relevant reviews (eg, on practice effects, composite scores).
2. **Interpretation**: The heterogeneous and multifactorial nature of cognitive tests offers particular challenges to their analysis by non‐specialist researchers. We summarize fundamental properties (such as practice/learning effects, cognitive domain specificity, and test‐function correspondence) to promote best practice for analyses involving cognitive test scores using statistical and computational approaches.
3. **Future directions**: Informed handling of cognitive data will promote more valid outcomes from analyses of large AD datasets, including robust analytical innovations, better study design, and evaluation of outcomes to benefit people touched by AD and other dementias.

### 2.2 Data‐collection initiatives

Various initiatives collect multicenter, multimodal, longitudinal data on AD and other types of dementia. Examples are: Alzheimer's Disease Neuroimaging Initiative (ADNI), Layton Aging & Alzheimer's Disease Center (LAADC), and National Alzheimer's Coordinating Center (NACC). Other projects focus on specific populations or cohorts, such as Vienna‐Trans‐Danube Aging study, and large biobank studies (eg, UK Biobank).

One of the most prominent in AD research is ADNI. ADNI was launched in 2004, as a longitudinal multicenter study funded by 20 companies (including pharma and non‐profit organizations), and other foundations, such as the National Institute on Aging (NIA), the Foundation for the National Institutes of Health (FNIH), and the Food and Drug Administration (FDA). The project aims to identify clinical, biochemical, genetic, and imaging markers to guide early detection of the disease and support treatment development, prediction of disease progression, and trajectory. Participants meeting eligibility criteria are recruited from various sites in North America.^8^ Other initiatives tackle similar challenges, and consequently collect similar data, with primary differences being the focus of study, for example, clinical, radiological, or biological.

We report on cognitive tests that are common among the protocols of the free access initiatives listed in Table 1. Building an exhaustive picture of all the cognitive tests used in AD clinical practice is outside the scope of this work, as it varies for each clinical context, location, and purpose of assessment. However, we report detailed information for measures and test batteries commonly featured in data‐sharing initiatives (Tables S1‐S6 in supporting information), assessment description, subscales, and scoring system.

**TABLE 1:** Overview on three main AD data collection initiatives

Name	Short description	Number of participants	Type of data collected	Cognitive tests
ADNI (University of California, SF)	Since 2004, ADNI collects longitudinal data from 58 sites in North America. The aim is to identify early biomarkers to support diagnosis and treatment development.	>3500	Clinical/cognitive assessments, medical/family history, neuroimaging (MRI, PET), biospecimen (plasma, CSF, metabolomic, proteomic), genetic (ApoE, GWAS/WGS data), neuropathology (1285 attributes)	MMSE, ADAS‐cog, MoCA, CFT, Clock Drawing, LM‐I, LM‐II, BNT/MINT, RAVLT, TMT A‐B, ANART.
LAADC (Oregon Health and Sciences University)	Dataset from the Layton Aging & Alzheimer's Disease Center, supported by the National Institute on Aging (NIA, NIH). Emphasis on studying preclinical and early dementia	1026	Clinical, MRI, and genetic data, as well as biological specimens. (486 attributes)	MMSE, CFT, BNT, LM‐I, LM‐II, TMT A‐B, Digit span, Digit symbol, Stroop task, CFL, WAIS Digit Span
NACC (NIA‐NIH)	The National Alzheimer's Coordinating Center was established in 1999 by 34 centres supported by U.S. National Institute on Aging/NIH.	35768	Clinical evaluations, neuropathology, MRI. (187 attributes)	MoCA, LM‐I, LM‐II, Benson Complex Figure copy, Digit Span, CFT, BCF recall, MINT, VF phonemic, TMT A‐B.

ABBREVIATIONS: ADAS‐cog, Alzheimer's Disease Assessment Scale–cognitive; ADNI, Alzheimer's Disease Neuroimaging Initiative; ANART, American National Adult Reading Test; ApoE, apolipoprotein E; BNT, Boston Naming Test; CFT, Category Fluency Test; CSF, cerebrospinal fluid; GWAS, genome‐wide association study; LAADC, Layton Aging & Alzheimer's Disease Center; LM, logical memory; MINT, multilingual gaming test; MMSE, Mini‐Mental State Examination; MoCA, Montreal Cognitive Assessment; MRI, magnetic resonance imaging; NACC, National Alzheimer's Coordinating Center; NIA, National Institute on Aging; NIH, National Institutes of Health; PET, positron emission tomography; RAVLT, Rey Auditory Verbal Learning Test; TMT A/B, Trail Making Test A‐B; VF, Verbal Fluency; WAIS, Wechsler Adult Intelligence Scale; WGS, whole genome sequencing.

Note: Each includes > 1000 participants, and > 100 attributes, including cognitive tests.

### 2.3 Contribution from data‐driven methods

**FIGURE 1:**
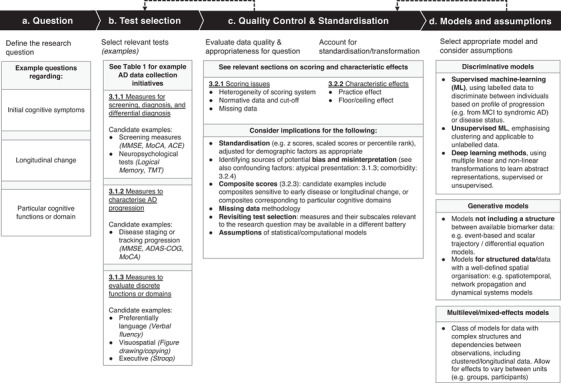
Flowchart representing example (A) research questions and steps regarding (B) test selection, (C) quality control and standardization, and (D) computational/statistical methods. Example research questions (A) correspond closely to test selection (B), while subsequent processes outlined in steps C and D are broadly relevant across questions and tests. Example measures are reported in italics and section headings underlined. Dashed arrows indicate revisiting steps, for example, revisiting test selection owing to missing data

Analyses afforded by data‐sharing initiatives may offer promise in complementing aspects of current, often qualitative, clinical practice. Data‐driven models have been developed intending to identify patterns from unlabeled data while requiring limited or no human input^9^ (for examples of discriminative, generative, and other generative approaches, see Figure 1). One example relevant to AD is the event‐based model (EBM), which combines various disease biomarkers into a quantitative signature of disease progression.^1^ Data‐driven methods have been used to identify subtypes or clusters in progression trajectories,^10^ or the fine‐grain temporal evolution of the disease.^11^ Examples of data‐driven approaches include identifying cognitively defined subgroups largely comprising typical, memory‐led, and atypical clusters, and comparing demographic and biological factors and prognosis between subgroups. Cognitive measures have been used to characterize and validate data‐driven subtypes identified through structural imaging,^10^, ^12^ partially predicated on well‐documented atypical exemplars of phenotypic heterogeneity, such as posterior cortical atrophy.^4^, ^7^ As with other statistical methods, models have different assumptions;^9^ these may include the assumption of a common disease trajectory across individuals and biomarker/test independence, which may be violated by clinical heterogeneity (typical versus atypical presentation) and dependency between tests and biomarkers, respectively.

## 3 PERSPECTIVES

Quantitative and qualitative methods are complementary for advancing our understanding of AD progression. However, quantitative research must maintain clinical relevance, which requires some domain knowledge that most data scientists do not have. This is particularly important with cognitive test data, being one of the primary markers used to track disease progression. In the following sections we present key considerations for selecting tests for inclusion in data‐driven modelling studies and for avoiding common misinterpretations. Sections are cross‐referenced in Figure 1, outlining example research questions and processes regarding test selection, quality control and standardization, and computational/statistical methods. We outline recent directions in cognitive assessment, and make suggestions for improving these data resources.

### 3.1 Considerations for test selection

Batteries of tests are generally rich and diverse, with correspondingly diverse options for tests to select as either input to a model or for validating a model. Taking into account the characteristics of different tests can support best use and more accurate contribution to knowledge (see Figure 1B). We focus on how different tests can be differentially sensitive at various stages of the disease, and additional considerations for tests particularly suited for certain analyses. Some tests are more appropriate for detecting early cognitive impairment, while others are more appropriate in assessing patients at intermediate/later disease stages or longitudinal change. This might be due to task difficulty, properties of tests, or composition of a test battery.

#### 3.1.1 Measures for screening, diagnosis, and differential diagnosis

Early diagnosis of AD continues to be a major challenge, requiring clinical tools sensitive to the most subtle changes that might emerge in the prodromal phase of AD, prior to clear impairment in everyday functioning. Commonly used screening measures include the Mini‐Mental State Examination^13^ (MMSE), Montreal Cognitive Assessment (MoCA), and Addenbrooke Cognitive Examination (ACE), though these should be used as support to a comprehensive clinical assessment, being screening tools and not diagnostic instruments.^13^ The MoCA may outperform MMSE in detecting early changes due to the disease: Freitas et al.^14^ found that MoCA is better at discriminating between mild cognitive impairment (MCI) or AD patients and healthy controls, and between MCI and AD, compared to MMSE. This might be due to the MoCA having increased focus on multiple cognitive domains (executive function, language, short‐term memory, or visuospatial skills). Individual neuropsychological measures with good sensitivity and specificity for distinguishing AD, MCI, and healthy control participants include the Logical Memory test,^15^ typically featuring immediate and delayed recall, and Trail Making Test (TMT) accuracy and time‐based measures of task‐switching/inhibition, working memory, and visuomotor ability.^16^

#### 3.1.2 Measures to characterize AD progression and evaluate moderate AD

MMSE and the Alzheimer's Disease Assessment Scale‐Cognitive subscale (ADAS‐Cog) are often used to stage and track AD progression during the symptomatic phase. Within these measures, MoCA may be more sensitive to longitudinal decline than the MMSE.^14^ ADAS‐cog is a commonly used indicator of disease progression in mild to moderate AD, though item‐level analyses suggest the ADAS‐Cog is most informative when administered to patients with moderate cognitive impairment,^17^ and individual subscore and rater items may be particularly sensitive to longitudinal change.^18^ For examples of challenges in appropriately selecting and interpreting tests across disease stages including practice, floor and ceiling effects, see section 3.2.2.

#### 3.1.3 Measures used to evaluate discrete functions or domains

Above neuropsychological tests (eg, Logical Memory, TMT), and composites thereof, are more appropriate for evaluating particular cognitive domains or functions. As mentioned above, test performance is multifactorial, for example, verbal fluency tasks place demands on both language and executive domains, and an individual's profile of performance should be considered rather than a single test. For examples of preferential assignment of subscales to domains and subdomains, see supporting information tables (Tables S1‐S6). If the purpose is to characterize cognitive domains, composite scores may mitigate individual test idiosyncrasies^3^ (see Cognitive Composites in section 3.2.3). Measures may be confounded in their interpretation when administered to certain patients, particularly those exhibiting prominent atypical non‐memory symptoms, eg, measures of executive function featuring prominent visual components being susceptible to visuospatial impairment.

### 3.2 Considerations in test analysis and interpretation

Cognitive test data variously depend on multiple factors such as the scoring system, the task, the domains that task is preferentially measuring, inter‐rater reliability, and numerous other elements related to individual characteristics and psychological status (fatigue and anxiety are good examples of this). Providing an exhaustive summary of these factors is outside the scope of this article. In this section we summarize considerations to minimize misinterpretation and misuse of cognitive data by computational scientists developing data‐driven predictive models of the disease.

#### 3.2.1 Scoring issues

**Heterogeneity of scoring systems**. Cognitive tests often differ in administration and scoring system, complicating the comparison of results across tests.^2^ In some cases, the direction of the scoring system might be counterintuitive, eg, optimal performance represented as a score of zero (ADAS‐Cog total items). Tests such as the TMT A/B record time taken to complete a task as a continuous measure, with long times corresponding to poor performance. This is also true for Digit Symbol and the number cancellation task in ADAS‐Cog. Other tests are scored using a defined ordinal scale, such as the Clock Drawing Test. Batteries such as the ADAS‐Cog 13‐item scale incorporate continuous, censored, and ordinal measures. While standard scores are routinely used to compare performance on different tests either within‐sample or relative to a normative sample, that skewed distributions (see below Floor or ceiling effects in section 3.2.2) complicate their interpretation. Tests might also include qualitative indicators such as “remembering test instructions,” “spoken language ability,” “word‐finding difficulty,” and “comprehension” in ADAS‐Cog. This heterogeneity in scoring systems may impede comparisons or integration with other measures and should be considered when planning analysis. Moreover, heterogeneity in the form of clinical presentation may add further complexity to the interpretation of individual test scores (see section 3.1.3).

**Normative data and cut‐off**. Normative data appropriately stratified by demographics enables determining cut‐off scores, the level at which a performance is considered impaired. Impaired performance is conventionally defined as below the fifth or first percentile based on normative data in clinical practice (for a summary of misapplying scores, see Della Sala and Cubelli^6^). Test‐dependent variations in performance are influenced by demographic factors including age, education, and sex, and care should be taken in using tests for which normative data are not available. Correspondingly, results from models trained on a certain normative sample should be handled carefully if used to gather insights on separate samples differing in demographic characteristics.^19^ The National Alzheimer's Coordinating Center Uniform Data Set provides an useful tool, offering neuropsychological scores adjusted for sex, age, and education.^20^ It is also important to note that if cognitive scores are already normalized for other factors (eg, age, education), then these factors should not be added a second time as covariates in a data‐driven model.

**Missing data**. It is important to understand possible causes of missing data, how to best interpret available information, and whether this affects only part of the assessment or its entirety. Various factors may underlie missing data or participants considered “untestable.” These include patients’ difficulty in complying with test instructions, particularly with more demanding tests and in patients with a greater degree of cognitive impairment and/or anxiety, premorbid language aptitude/literacy, comorbidity and uncorrected sight or hearing loss, or time constraints. Determining whether data are missing completely at random, missing at random, or missing not at random is reliant on establishing factors underlying missing data through available records. While many approaches assume data are missing at random, this assumption is often violated for cognitive data where participants may be unable to complete tests owing to degree of impairment. For a summary of types of missing data and statistical methods to handle missing data including random effects models, Bayesian approaches, inverse probability weighting, and imputation, see Sterne et al.^21^ It is worth noting that some subscales overlap across batteries, so a missing subscale might be available in a different battery for the same participant.

#### 3.2.2 Characteristic effects of cognitive tests

**Practice effect**. One of the main uses of cognitive tests is the repeated administration for tracking progression, for example, in clinical trials. It is therefore vital to be aware of practice effects, defined as “the improvement in serial cognitive tests with the same or similar test materials.”^22^ Such effects may be particularly evident on measures of episodic memory, between initial retesting, diminishing across subsequent visits, and in MCI and AD patients as well as healthy participants.^23^ It can also substantially alter interpretation of findings with inadequate control or inappropriate analysis. To overcome this limitation, many cognitive tests have validated alternative forms administered in a counterbalanced order, although there is evidence that they only attenuate and do not eliminate the effect.^24^ Goldberg et al.^25^ suggest three different approaches to attenuate the consequences of practice effect with varying advantages and disadvantages: introducing massed practice to increase task familiarity, adopting cognitive science principles to reduce practice‐related gains, and developing well‐matched alternate forms. While the above efforts are intended to mitigate practice effects, there is increasing evidence on the clinical utility of characterizing practice effects themselves, for example in determining their associations with AD risk factors and biomarkers, or predicting subsequent cognitive decline.^26^, ^27^

**Floor or ceiling effects**. These occur when the test cannot measure performance outside the test range, which overestimates or underestimates performance and skews score distributions. This is a common issue with brief cognitive tests that measure a limited range of task performance. Patients, particularly at an early disease stage, may make few or no errors on common tests, such as MMSE or ADAS‐Cog.^28^, ^29^ A key challenge is selecting tests on which patients at intermediate disease stages might perform adequately, while being of sufficient difficulty to be sensitive for high‐functioning patients and healthy control participants. Tests meeting such criteria might still yield variability in task performance that differs considerably between patients and healthy controls, or between patient groups stratified by severity. Although not all measures are susceptible to floor and ceiling effects,^30^ many cognitive tests used for computational purposes might need further analysis or subscales selection^31^ before comparing them to other markers. Approaches that are less prone to floor or ceiling effects include tests whose measurement characteristics include both accuracy and timed components, tests without a fixed maximum score, and experimental designs not featured in data initiatives (eg, using a staircase paradigm) or composite measures.^3^

#### 3.2.3 Cognitive composites

There is a recent surge in composites derived from batteries of tests in AD research.^5^ They have been developed for multiple purposes, including sensitivity to global disease severity,^32^ individual cognitive domains,^2^, ^3^ or longitudinal change^33^—particularly in the preclinical phase relevant to secondary prevention trials.^34^ In their recent review, Schneider and Goldberg^5^ identified 12 composite scales that have been used in clinical trials to assess cognitive functions. Multi‐domain composites may mitigate previously discussed inability to isolate single domains, and may be sensitive to domains that are affected in the preclinical stages of the disease.^34^ Various methods have explored composite development, such as psychometric;^3^ a combination of statistical, theoretical, and empirical approaches;^33^ and computationally sophisticated data‐driven algorithms.^35^ However, cognitive composites are still prone to a number of issues.^5^ Lim et al.^36^ mention the importance of evaluating the sensitivity of each scale contributing to the composite, as it can affect the overall sensitivity of the composite. Moreover, domains relevant to early clinical symptoms of AD are often underrepresented.^5^ For example, while episodic memory deficits are one of the earliest and best recognized indicators of preclinical AD, non‐memory domains may also be susceptible to pathological changes during the preclinical phase. Overall, a cognitive‐composite approach might be appropriate in clinical trials and disease progression monitoring,^37^ but current measures face various limitations in their validation and psychometric assumptions.^5^

#### 3.2.4 Considering comorbidity and other factors

Comorbidities can confound cognitive test scores. Notably, depression and anxiety are known to have strong effects on cognitive performance. For example, Qiu et al.^38^ reported depression contributing to cognitive dysfunction in mild to moderate AD, highlighting a need to handle cognitive test results carefully and consider various factors in their interpretation. Other factors include native language, literacy,^39^ and uncorrected sensory loss. This reinforces the importance of considering cognitive tests in the context of behavioral and other clinical examinations. In some cases, the qualitative experience of a patient during a quantitative assessment is recorded and could support the interpretation for missing data or the psychological/behavioral status of the interviewee during the assessment.

## 4 FUTURE DIRECTIONS

Big data initiatives have already contributed to a better understanding of AD and its characteristics. However, there are still ways in which these resources can be further developed. First, by highlighting the challenges of creating resources that enable a comprehensive representation of the AD spectrum. Current large cognitive datasets are broadly characterized by an over‐representation of tests preferentially reflecting certain functions (memory) more so than others (visual, motor). This risks imposing constraints on appreciating the range and basis of AD phenotypic heterogeneity, not only regarding canonical atypical clinical phenotypes associated with AD,^4^ but also in typical late onset AD.^2^ Furthermore, while memory issues are generally among the first AD markers, there is evidence that other cognitive functions may be sensitive to early detection of the disease (eg, spatial navigation deficits^40^). Examples such as the Dominantly Inherited Alzheimer Network (DIAN) study^41^ demonstrate how trials can adapt to incorporate additional measures according to advances in research.

One important step toward the adaptation of cognitive assessments for computational use is digital tests^37^, whether comprising paper‐based tests converted into a digital form or novel testing paradigms. Examples are Cambridge Neuropsychological Test Automated Battery (CANTAB)^42^ or the Cogstate battery.^43^ One particular feature of most web‐based testing is self‐administration, which gives users the opportunity to complete the test remotely, at their own pace, and does not require additional hardware or software download.^44^ More recently we see the advent of tests using eye‐tracking technology^45^ frequently embedded in serious games or in augmented reality/virtual reality systems.^46^ This approach overcomes the possible language barrier, both regarding instructions and verbal responses,^47^ giving the possibility for computational methods to identify subtle biomarkers for early disease detection and progression.^45^

Digital tests have numerous potential advantages. Not only scoring, but also detailed reaction times and behavioral measures are recorded in a standardized manner. Automatic development of datasets and recording of repeated measures may facilitate data storage, saving time and costs for analysis. One interesting advantage is the development of adaptive computerized tests, which have promise in mitigating floor and ceiling effects^48^ while accommodating effective counterbalancing. Computerized tests offer opportunities to efficiently compare individuals against a population, and may offer scalable measures to determine abnormal performance currently evaluated qualitatively based on experience‐led judgments, for example quantitatively evaluating speech patterns from audio recording of verbal fluency. While these examples refer to administered or self‐administered cognitive assessments, digital markers of behaviors not requiring task engagement^47^ increasingly evaluate speech detection features, physiological measures, and activity, ultimately intending to promote ecological, continuous assessment.^49^ Despite its potential, the uptake of this technology is still slow compared to the classic examinations. This might be due to limited validation, insufficient normative data, and issues around technology access and harmonization.^49^ Improvement in this area will not only reinforce the collaboration between disciplines, but provide consistent sources of data and patient monitoring, hopefully leading to better early detection and understanding of the disease.

Finally, the adoption of data‐driven models in healthcare, many of which may be considered “black box” in nature, has received a number of criticisms. General concerns regarding interpretability of machine learning and artificial intelligence algorithms are arguably particularly relevant in clinical applications, where results can influence clinical decisions and health outcomes and present unique ethical challenges.^50^ Interpretability touches on all stages of the development and use of these models, including the dataset used, the explainability of the models’ decision, and the interpretation of results according to domain knowledge. Regarding datasets themselves, selection and other biases in their composition must be acknowledged along with their implications for interpreting findings. Understanding of models’ decisions is of particular importance in establishing replicability and generalizability of results. While limitations in understanding are often contextualized within trade‐offs between their explainability and performance, there are increasing efforts to explain model decisions and results, for example based on presenting model features with observed behavioral data.^47^ Regarding interpretation based on domain knowledge, nominally significant results do not necessarily constitute clinically meaningful or informative findings at the population, group, or individual level. To promote relevance of analyses across clinical and research contexts, involving clinicians and researchers with domain expertise in interpreting cognitive test data offers key contributions in formulating research questions, planning analyses, and interpreting findings.

### 4.1 CONCLUSIONS

Research in AD is moving toward increasing collaboration between disciplines to better understand and address this condition. The creation and sharing of big datasets are important vehicles guiding this effort in the coming years. In particular, cognitive measures are currently one of the most used quantitative methods in clinical practice, although not necessarily familiar to non‐clinical disciplines. We have intended to promote understanding and address knowledge gaps around use and misuse of cognitive tests for a broad audience of researchers from different fields. Ultimately, we hope that better appreciation of the promises and applications of cognitive data will stimulate timely interdisciplinary advances in our understanding of AD.

## FUNDING INFORMATION

This work is supported by the EPSRC CDT in Medical Imaging (EP/L016478/1) and by the industrial partner icometrix (https://icometrix.com). This project has received funding from the European Union's Horizon 2020 research and innovation programme under grant agreement No. 666992, and by the EPSRC grant EP/M020533/1. NPO is a UKRI Future Leaders Fellow (MR/S03546X/1). KXXY is funded by the Alzheimer's Society, grant number 453 (AS‐JF‐18‐003).

## FINANCIAL DECLARATIONS

Nothing to declare.

## CONFLICTS OF INTEREST

The authors declare that they have no conflicts of interest.

## Supporting information

Supplementary material
