# Supplementary material for: Analyzing large Alzheimer's disease cognitive datasets: Considerations and challenges
Source: Alzheimers Dement (Amst). 2020 Dec 7;12(1):e12135. doi: 10.1002/dad2.12135 (PMC7720865; doi:10.1002/dad2.12135)
Supplement: Supplementary file 1 — Supplementary material [file DAD2-12-e12135-s001.docx]

## Supplementary material

# **Table 1 - Overview on a set of commonly used cognitive tests for AD.**

| **Test** | **Short Description** | **Subscales** |
| --- | --- | --- |
| Alzheimer’s Disease Assessment Scale – cognitive (ADAS-cog)^1^ | Brief cognitive battery, with items intended  to evaluate different cognitive domains (in particular memory, language, praxis, and orientation). | 13 |
| Mini-Mental State Examination (MMSE)^2^ | Brief screening and staging instrument for  mild cognitive dysfunction. Mainly evaluating orientation, attention, working and short term memory, language, and constructional praxis. | 10 |
| Montreal Cognitive Assessment (MoCA)^3^ | Brief screening instrument assessing various  cognitive domains | 11 |
| Ray Auditory Verbal Learning Task (RAVLT)^4^ | Test of episodic memory, immediate recall or  delayed recall | 9 |
| Boston Naming Test (BNT)^5^ | Visual naming test | - |
| Category Fluency (CF)^6^ | Word fluency test according to a specified category of names, to test semantic memory and word recall. | - |
| Clock Drawing Test (CDT)^7^ | Test of visuo-constructional abilities | - |
| Trail Making Test (TMT) A / B^8^ | Test of visual attention, psychomotor speed and task switching, in two parts. | 2 |
| Logical Memory (LM) I-II^9^ | Learning test of a short story, immediate and  delayed. | 2 |

# **Table 2 - ADAS-cog: subscales and description**

| **Item** | **Subscale** | **Task**  (*examiner rated item) | **Range**  [max - min] | **Scoring** | **Domain** |
| --- | --- | --- | --- | --- | --- |
| Q1 | Word Recall | Three trials to learn as many | [0-10] | Mean N of recalled words in the | Memory |
|  |  | high-frequency words |  | three trials |  |
| Q2 | Commands | Performance of 5 separate com- | [0-5] | Steps correctly performed | Language |
|  |  | mands involving 1 to 5 steps per |  |  |  |
|  |  | command |  |  |  |
| Q3 | Constructional Praxis | Copy of 4 geometric forms with increasing level of difficult (circe, overlapping rectangles, diamond, cube). | [0-5] | Score is based on the number of correctly copied forms | Visuospatial functions, Executive functions |
| Q4 | Delayed Word Recall | Free recall of the word list in | [0-10] | Mean N of recalled words in the | Memory |
|  |  | Q1, approximately 5 minutes af- |  | three trials |  |
|  |  | ter trial 3 of the Q1 |  |  |  |
| Q5 | Naming Task | 1. Name 12 drawn objects with | [0-5] | 5 ranges of scores, with 0 being | Language |
|  |  | high/medium/low frequency; 2. |  | the best performance |  |
|  |  | Name the fingers of the dominant |  |  |  |
|  |  | hand |  |  |  |
| Q6 | Ideational Praxis | Performance of a sequence of ac- | [0-5] | Scoring based on correctly per- | Apraxia |
|  |  | tions participants would do in or- |  | formed components of the task |  |
|  |  | der to send a letter to themselves |  |  |  |
|  |  | (fold letter, put in envelope, seal, |  |  |  |
|  |  | address and put a stamp on it). |  |  |  |
| Q7 | Orientation | Questions regarding orientation | [0-8] | Number of correct responses | Orientation |
|  |  | to time and place |  |  |  |
| Continued on next page | | | | | |

**Table 2: ADAS-cog: subscales and description (continued from previous page)**

| **Item** | **Subscale** | **Task** | **Range** | **Scoring** | **Domain** |
| --- | --- | --- | --- | --- | --- |
| Q8 | Word Recognition | After reading a list of 12 words,  participants have to recognise these words from a new list were including both old and new words. It comprises three trials Examiner’s assessment of the par- ticipant’s ability to remember test instructions  Examiner’s assessment of the par- ticipant’s ability to understand speech  Examiner’s assessment of the par- ticipant’s ability in word-finding Examiner’s assessment of the par- ticipant’s ability to communicate verbally  Selective cancellation of target numbers on a page (timed: 45 seconds) | [0-12] | Mean N of recalled words in the  three trials | Memory |
| Q9  Q10 | Remembering Test In- structions  Comprehension |  | [0-5]  [0-5] | Examiner’s assessment (0 = no difficulties; 1 = very mild; 2 = mild; 3 = moderate; 4 = moder- ately severe; 5 = severe) Examiner’s assessment |  |
| Q11 Q12 | Word Finding Diffi- culty  Spoken Language Ability |  | [0-5]  [0-5] | Examiner’s assessment Examiner’s assessment |  |
| Q13 | Number Cancellation |  | [0-40] | [N of correctly crossed items in 45 seconds] – [N of incorrectly crossed items] – [reminders] | Visuospatial/Attention |

**Table 3 - MMSE: subscales and description**

| **Item** | **Subscale** | **Task** | **Range**  [min - max] | **Scoring** | **Domain** |
| --- | --- | --- | --- | --- | --- |
| Q1 | Orientation | Questions to assess patient’s ori- | [0-10] | 1 point for each correct answer | Orientation |
|  |  | entation in space and time |  |  |  |
| Q2 | Immediate Recall | Three common words are read | [0-3] | Number of correct words in the | Memory |
|  |  | and patients have to repeat them |  | correct position |  |
|  |  | in the same order |  |  |  |
| Q3 | Attention | Patients are asked to subtract 7 | [0-5] | Scoring is given by the number | Attention / Executive |
|  |  | from 100 for 5 times. Alterna- |  | of digits within the patients re- | functions |
|  |  | tively, they are asked to spell a |  | sponse that has the most ascend- |  |
|  |  | word of 5 letters backward |  | ing digits. |  |
| Q4 | Delayed Recall | Recall the three words in Q2 | [0-3] | Number of correct words in the | Memory |
|  |  |  |  | correct position |  |
| Q5 | Naming Task | Patients are asked to name a | [0-2] | Number of correct responses | Language |
|  |  | wristwatch and a pencil, when |  |  |  |
|  |  | shown to them |  |  |  |
| Q6 | Command | Patients have to execute a com- | [0-3] | 1 point for each correctly per- | Executive functions, |
|  |  | mand that implies a sequence of |  | formed action | Language |
|  |  | 3 actions |  |  |  |
| Q7 | Repetition | The examiner reads a sentence | [0-1] | 1 point when the sentence is cor- | Language |
|  |  | and patients have to repeat it |  | rectly repeated |  |
| Q8 | Reading | Patients have to read a sentence | [0-1] | 1 point if the instructions are |  |
|  |  | shown on a paper sheet and per- |  | completely and correctly per- |  |
|  |  | form the action |  | formed |  |
| Q9 | Writing | Patients are asked to write a sen- | [0-1] | 1 point for a semantically and |  |
|  |  | tence they choose |  | grammatically correct sentence |  |
| Continued on next page | | | | | |

**Table 3: MMSE: subscales and description (continued from previous page)**

| **Item** | **Subscale** | **Task** | **Range** | **Scoring** | **Domain** |
| --- | --- | --- | --- | --- | --- |
| Q10 | Construction | Patients have to copy the design  of two intersecting pentagons | [0-1] | The figures should have five an-  gles each and intersect to score 1 point | Visuospatial |

**Table 4 - MoCA: subscales and description**

| **Item** | **Subscale** | **Task** | **Range**  [min - max] | **Scoring** | **Domain** |
| --- | --- | --- | --- | --- | --- |
| Q1 | Trails | Draw a line (1) connecting num- | [0-1] | Correct if the sequence is correct | Executive functions |
|  |  | bers in ascending order (2) alter- |  | / autocorrected | Visuospatial |
|  |  | nating one number and one letter |  |  |  |
|  |  | in ascending order |  |  |  |
| Q2 | Cube | Three common words are read | [0-1] | Specific criteria to assess this item | Visuospatial |
|  |  | and patients have to repeat them |  |  | Executive functions |
|  |  | in the same order |  |  |  |
| Q3 | Clock | Draw a clock with all numbers, | [0-3] | Criteria: contour, numbers, | Visuospatial |
|  |  | then set the time to 11:10 |  | hands | Executive functions |
| Q4 | Naming | Tell the name of drawn animals | [0-3] | Number of correct items | Language |
| Q5 | Memory Immediate | Read a list of 5 works and recall | [0-5] | Number of words correctly re- | Memory |
|  |  | them afterwards |  | called at the first trial |  |
| Q6 | 1. Digit Span Forward | Repeat a sequence of 5 digits (1 | [0-1] | 1 point if performed correctly | Executive functions |
|  | (attention) | digit/sec) |  |  |  |
|  |  |  |  |  |  |
|  | 2. Digit Span Back- | Repeat a sequence of 3 digits backwards (1 | [0 - 1] | 1 point if performed correctly | Executive functions |
|  | wards (attention) | digit/sec) |  |  |  |
|  | 3. Letters and tapping | The examiner reads a list of let- | [0 - 1] | No point if more than 2 errors | Executive functions |
|  | (vigilance) | ters, the participant has to tap |  |  |  |
|  |  | anytime the letter A is read |  |  |  |
|  | 4. Serial 7s (vigilance) | The patient is asked to subtract | [0 - 3] | 4 or 5 correct = 3pt; 2/3 correct | Executive functions |
|  |  | 7 from 100 and other 7 until the |  | = 2pt; 1 correct = 1pt |  |
|  |  | stop command |  |  |  |
| Continued on next page | | | | | |

**Table 4: MoCA: subscales and description (continued from previous page)**

| **Item** | **Subscale** | **Task** | **Range** | **Scoring** | **Domain** |
| --- | --- | --- | --- | --- | --- |
| Q7  Q8  Q9 Q10  Q11 | Sentence Repetition  Verbal Fluency  Abstraction Delayed Recall  Orientation | The examiner reads a sentence  and patients have to repeat it (2 sentences)  Name the maximum amount of works beginning with F (time: 1 min)  Tell how two items are alike Recall the words in Q5  Questions to assess the orienta- tion of patients in space and time | [0-2]  [0- max named in 60s]  [0-2]  [0-5]  [0-6] | 1 point correctly repeated sen-  tence  Total number of words, minus any intrusions, duplication, or other errors  1 trial and 2 counted trials Criteria: recall with no cue, cate- gory cue, multiple choice cue Number of correct responses | Language  Phonemic fluency  Semantics Memory  Orientation |

**Table 5 - RAVLT: subscales and description**

| **Item** | **Subscale** | **Task** | **Range**  [min - max] | **Scoring** | **Domain** |
| --- | --- | --- | --- | --- | --- |
| 1 | List A: Trial I | The examiner reads a list of 15  words and the participant has to recall as many as possible  The examiner reads the same list of 15 words and the participant has to recall them  The examiner reads a new list of 15 words and the participant has to recall as many as possible  The participant has to recall as many words as possible from List A  After 30 minutes. the participant has to recall a many words as possible from List A  The participant has to recognise as many words as possible from a list mixing words from List A with new words | [0 - 15] | Number of recalled words (num-  ber of intrusions is also counted) | Episodic memory |
| 2 to 5 | Trial II to V |  | [0 - 15] | (as above) | Episodic memory |
| 6 | List B |  | [0 - 15] | (as above) | Episodic memory |
| 7 | Trial VI |  | [0 - 15] | Number of correct items | Episodic memory |
| 8 | 30 min delayed |  | [0 - 15] | (as above) | Episodic memory |
| 9 | Recognition |  | [0 - 15] | Number of recognised words (number of intrusions is also counted) | Episodic memory |

**Table 6 - Other tests: subscales and description**

| **Item** | **Subscale** | **Task** | **Range**  [min - max] | **Scoring** | **Domain** |
| --- | --- | --- | --- | --- | --- |
|  |  | The patient has to name 30 images  The patient has to name as many items from an assigned category as possible (animals, vegetables, time: 60 sec each)  The patient is asked to (1) draw a clock and the time; (2) copy a clock and the time   1. The examiner reads a story and the patient has to recall it. 2. Delayed recall of the story 3. Connect numbered circles in ascending numerical order 4. Connect circles with numbers and letters in alternate order |  | Number of correctly recognised items (sum of uncued + seman- tic + phonemic cues)  Number of correct responses  Score according to the number of criteria met  Number of chunks of information correctly recalled  Time to complete the task (A. max 150 sec; B. max 300 sec) Number of commissions or omis- sion errors |  |
| BNT | Boston Naming test |  | [0 - 30] |  | Language |
| CF | Word fluency test |  | [0- max named in 60s] |  | Semantic fluency |
| CDT | Constructional ability |  | [0 - 5] |  | Executive functions, visuospatial functions |
| LM I/II | Episodic memory |  | [0 - 25] |  | Episodic memory |
| TMT  A/B | Trail Making Test |  | [time]  N omissions  N commissions |  | Executive functions, visuospatial functions |

## REFERENCES

1. Rosen WG, Mohs RC, Davis KL. A new rating scale for Alzheimer’s disease. *Am J Psychiatry*. 1984;141(11 (1356-1364),). doi:10.1176/ajp.141.11.1356

2. Folstein MF, Robins LN, Helzer JE. The Mini-Mental State Examination. *Arch Gen Psychiatry*. 1983;40(7):812. doi:10.1001/archpsyc.1983.01790060110016

3. Nasreddine ZS, Phillips NA, Bédirian V, et al. The Montreal Cognitive Assessment, MoCA: A Brief Screening Tool For Mild Cognitive Impairment. *J Am Geriatr Soc*. 2005;53(4):695-699. doi:10.1111/j.1532-5415.2005.53221.x

4. Rosenberg SJ, Ryan JJ, Prifitera A. Rey auditory‐verbal learning test performance of patients with and without memory impairment. *J Clin Psychol*. 1984;40(3):785-787. doi:10.1002/1097-4679(198405)40:3<785::AID-JCLP2270400325>3.0.CO;2-4

5. Mack WJ, Freed DM, Williams BW, Henderson VW. Boston Naming Test: Shortened Versions for Use in Alzheimer’s Disease. *J Gerontol*. 1992;47(3):P154-P158. doi:10.1093/geronj/47.3.p154

6. Caramelli P, Carthery-Goulart MT, Porto CS, Charchat-Fichman H, Nitrini R. Category Fluency as a Screening Test for Alzheimer Disease in Illiterate and Literate Patients. *Alzheimer Dis Assoc Disord*. 2007;21(1):65-67. doi:10.1097/WAD.0b013e31802f244f

7. Sunderland T, Hill JL, Mellow AM, et al. Clock Drawing in Alzheimer’s Disease: A Novel Measure of Dementia Severity. *J Am Geriatr Soc*. 1989;37(8):725-729. doi:10.1111/j.1532-5415.1989.tb02233.x

8. Reitan RM. Validity of the Trail Making Test as an Indicator of Organic Brain Damage. *Percept Mot Skills*. 1958;8(3):271-276. doi:10.2466/pms.1958.8.3.271

9. Abikoff H, Alvir J, Hong G, et al. Logical memory subtest of the Wechsler Memory Scale: age and education norms and alternate-form reliability of two scoring systems. *J Clin Exp Neuropsychol Off J Int Neuropsychol Soc*. 1987;9(4):435-448. doi:10.1080/01688638708405063
